# Pulmonary Circulation Transvascular Fluid Fluxes Do Not Change during General Anesthesia in Dogs

**DOI:** 10.3389/fphys.2018.00124

**Published:** 2018-02-21

**Authors:** Olga Frlic, Alenka Seliškar, Aleksandra Domanjko Petrič, Rok Blagus, George Heigenhauser, Modest Vengust

**Affiliations:** ^1^Veterinary Faculty, University of Ljubljana, Ljubljana, Slovenia; ^2^Institute for Biostatistics and Medical Informatics, University of Ljubljana, Ljubljana, Slovenia; ^3^Department of Medicine, McMaster University Medical Centre Hamilton, Hamilton, ON, Canada

**Keywords:** general anesthesia, pulmonary circulation, transvascular fluid flux, pulmonary edema, starling forces, Jacobs Stewart cycle, furosemide

## Abstract

General anesthesia (GA) can cause abnormal lung fluid redistribution. Pulmonary circulation transvascular fluid fluxes (*J*_*VA*_) are attributed to changes in hydrostatic forces and erythrocyte volume (EV) regulation. Despite the very low hydraulic conductance of pulmonary microvasculature it is possible that GA may affect hydrostatic forces through changes in pulmonary vascular resistance (PVR), and EV through alteration of erythrocyte transmembrane ion fluxes (_*ion*_*J*_*VA*_). Furosemide (Fur) was also used because of its potential to affect pulmonary hydrostatic forces and _*ion*_*J*_*VA*_. A hypothesis was tested that *J*_*VA*_, with or without furosemide treatment, will not change with time during GA. Twenty dogs that underwent castration/ovariectomy were randomly assigned to Fur (*n* = 10) (4 mg/kg IV) or placebo treated group (Con, *n* = 10). Baseline arterial (BL) and mixed venous blood were sampled during GA just before treatment with Fur or placebo and then at 15, 30 and 45 min post-treatment. Cardiac output (Q) and pulmonary artery pressure (P_AP_) were measured. *J*_*VA*_ and _*ion*_*J*_*VA*_ were calculated from changes in plasma protein, hemoglobin, hematocrit, plasma and whole blood ions, and Q. Variables were analyzed using random intercept mixed model (*P* < 0.05). Data are expressed as means ± SE. Furosemide caused a significant volume depletion as evident from changes in plasma protein and hematocrit (*P* < 0.001). However; Q, P_AP_, and *J*_*VA*_ were not affected by time or Fur, whereas erythrocyte fluid flux was affected by Fur (*P* = 0.03). Furosemide also affected erythrocyte transmembrane K^+^ and Cl^−^, and transvascular Cl^−^ metabolism (*P* ≤ 0.05). No other erythrocyte transmembrane or transvascular ion fluxes were affected by time of GA or Fur. Our hypothesis was verified as *J*_*VA*_ was not affected by GA or ion metabolism changes due to Fur treatment. Furosemide and 45 min of GA did not cause significant hydrostatic changes based on Q and P_AP_. Inhibition of Na^+^/K^+^/2Cl^−^ cotransport caused by Fur treatment, which can alter EV regulation and *J*_*VA*_, was offset by the Jacobs Stewart cycle. The results of this study indicate that the Jacobs Stewart cycle/erythrocyte Cl^−^ metabolism can also act as a safety factor for the stability of lung fluid redistribution preserving optimal diffusion distance across the blood gas barrier.

## Introduction

Transvascular fluid fluxes in the pulmonary circulation (*J*_VA_) may influence the diffusion distance between the pulmonary capillary and the alveoli, and compromise or improve gas exchange when lungs function undergoes physiological (i.e., exercise) or pathological adaptations (inflammation) (Vengust et al., 2013; Apostolo et al., 2014). Transvascular fluid fluxes in the pulmonary circulation are traditionally attributed to changes in hydrostatic forces and perfused alveolar capillary surface area, which are determined by the rise in mean pulmonary artery pressure (P_PA_) (Coates et al., 1984; Sinha et al., 1996; Vengust et al., 2006a). Erythrocyte volume (EV) regulation has also been associated with *J*_*VA*_ through transmembrane/transvascular ion redistribution (*Ion*_*VA*_) and intracellular osmolality changes (Wickerts et al., 1992; Vengust et al., 2006a, 2013).

General anesthesia (GA) is a critical event, with a potential to change pulmonary vascular resistance (PVR) (hydrostatic forces) through alterations in cardiac output (Q) and pulmonary blood flow (Fischer et al., 2003). It affects ventilation-perfusion (V/Q) matching, which triggers a variable degree of the hypoxic pulmonary vasoconstriction (HPV) to match regional ventilation and perfusion and to maintain oxygenation. HPV increases PVR; higher pressures at the microvascular level then lead to greater transmural hydrostatic driving gradients and increased *J*_VA_ (Starling, 1896; Fischer et al., 2003).

Transvascular fluid fluxes in healthy individuals at rest (steady state) are at or near zero (Coates et al., 1984; Newman et al., 1988; Schaffartzik et al., 1992; Wickerts et al., 1992; Vengust et al., 2006a, 2011, 2013). Several gravimetric studies identified an edemagenic P_AP_, which overcomes the hydraulic conductance of the lung microvasculature and the ability of pulmonary lymphatics to remove fluid from the lung parenchyma (Wickerts et al., 1992; Hoeper, 2009). Pulmonary microvasculature is very resilient to abnormal transvascular fluid redistribution (Schneeberger and Karnovsky, 1976; Bhattacharya, 1988; Maggiorini et al., 2001; Parker et al., 2006; Effros and Parker, 2009). However, *J*_*VA*_ during GA may still progressively become abnormal at sub edemagenic levels of P_AP_ (Chapman et al., 2005), and eventually compromise the diffusion distance across the blood gas barrier.

The purpose of this study was to investigate *Ion*_*VA*_ and *J*_*VA*_ during GA, which has not been investigated to date. Vengust et al. (2006a,b, 2011, 2013) in a series of investigations in exercising horses employed the method, which can detect *in vivo* changes in *J*_*VA*_, and even follow *J*_*VA*_ changes with time after the application of different therapeutic agents. Furosemide was used in this study to evaluate pulmonary circulation adaptations to changes in hydrostatic forces and/or ion fluxes (Mukherjee et al., 1981; Narins and Chusid, 1986; Boles Ponto and Schoenwald, 1990). Furosemide is used to treat abnormal lung fluid redistribution (lung edema) mostly due to its diuretic effect, but also the possibility to alter transmembrane ion fluxes through attenuation of Na^+^/K^+^/2Cl^−^ cotransport (Dikshit et al., 1973; Kracke and Dunham, 1987; O'Donnell, 1993). However, hydrostatic force increase and transvascular ion metabolism changes would have to be substantial to overcome the ability of pulmonary vasculature to keep pulmonary transvascular fluid dynamics at a steady state level (Schneeberger and Karnovsky, 1976; Bhattacharya, 1988; Maggiorini et al., 2001; Parker et al., 2006). Therefore, we tested the hypothesis that *J*_*VA*_, with or without furosemide treatment, will not change with time during GA.

## Methods

### Ethical approval

The study protocol was approved by the National Ethics Committee (document No.: U34401-23/2013/6), according to the relevant Slovene and European Union regulations.

Twenty dogs (10 males, 10 females), with a mean age of 26.3 months (range 11–65 months), mean weight of 29.35 kg (16.3–47.9 kg) were used. Gender was equally distributed between groups. The study was conducted while dogs underwent a routine castration or ovariectomy under GA. All dogs were classified as ASA I (healthy dogs without recognizable signs of disease) according to the American Society of Anesthesiologists. An informed client consent was obtained before the dogs entered the study.

### Experimental protocol

Dogs were randomly assigned to Fur (4 mg/kg IV) or placebo (Con) (0.9% saline solution at a volume corresponding to the Fur treatment IV) treatment. Dogs were premedicated with morphine (0.3 mg kg^−1^ SQ; Morfin Alkaloid, Alkaloid Skopje, FYROM). An intravenous catheter (BD Venflon, Becton Dickinson Infusion Therapy AB, Helsingborg, Sweden) was inserted into the left or right cephalic vein. Anesthesia was induced with midazolam (0.1 mg kg^−1^ IV; Midazolam Torrex, Chiesi -Pharmaceuticals GmbH, Austria), followed by propofol (3–4 mg kg^−1^ IV; Norofol, Norbrook Laboratories Limited, Northern Ireland). After endotracheal intubation, anesthesia was maintained with sevoflurane (Sevorane, AbbVie, Campoverdedi Aprilia, Italy) delivered in oxygen using a circle breathing system. Dogs were kept in dorsal position throughout the experiment to reduce variations in the distribution of blood flow and ventilation in the lung (Galvin et al., 2007). The electrocardiogram, end-tidal CO_2_ tension, and arterial oxygen saturation were monitored during anesthesia (BLT M9000 VET). Lactated Ringer's solution (5 mL kg^−1^ h^−1^ IV; B. Braun, Germany) was infused during the experiment.

A Swan-Ganz catheter (Baxter Healthcare Corp., Irvine, CA, USA) was placed via the left or right jugular vein into the pulmonary artery for mixed venous blood sampling, pulmonary artery pressure (P_AP_) and core body temperature measurement. Correct catheter placement was ascertained by observing characteristic pressure waveforms (HP Model 66S, Hewlett-Packard Company, Palo Alto, Calif.). Cardiac output was measured by the thermodilution technique (10 mL of 0.9% NaCl; injectate temperature, 23 to 25°C). Injectate volume and temperature were used according to the manufacturer's instructions (HP Component monitoring system anesthesia/standard; Ganz et al., 1971; Nemec et al., 2003).

### Blood sampling and analysis

Baseline (BL) arterial and mixed venous blood were sampled simultaneously just before treatment with Fur or Con and at 15, 30, and 45 min post-treatment. Surgery (castration/ovariectomy) then commenced within few min after the 45 min sample was taken. Blood samples were collected into lithium-heparinized syringes (Gaslyte, arterial blood sampler, Vital Signs, Inc., Englewood, CO, USA) and analyzed immediately in duplicates with the Rapid Point 500 analyzer (Siemens Healthcare, Erlangen, Germany). Rapid Point 500 uses ion selective electrode method (potentiometry) for the determination of electrolyte activity, including PCO_2_ (potentiometry based on Severinghaus). It uses amperometric oxygen electrode for PO_2_. Total hemoglobin (Hb) is determined by multiwavelength spectrophotometry. The analyzer automatically calibrates sensors several times a day. Intra-and inter- assay coefficients of variation for Rapid Point 500 have coefficients of determination (CV) higher than 0.91 (Nicolas et al., 2013), whereas for variables included in this study coefficients of determination was higher than 0.96. Hematocrit (Hct) was measured using microhematocrit method (CV = 0.96). Total plasma protein (PP) was measured using a clinical refractometer (Attago 331; Attago, Tokyo, Japan) (CV = 0.92). For whole blood [Na^+^], [K^+^], and [Cl^−^] determination, blood samples were repeatedly frozen (−80°C) and thawed (room temperature) to induce red cell lysis.

### Calculations

Calculation methods have been reported previously (Vengust et al., 2006a, 2011, 2013). Plasma volume changes across the lung (ΔPV) were calculated from changes in PP at the same time point from central venous to arterial blood according to Dill and Costill (1974). Changes in erythrocyte volume (ΔEV) across the lungs were calculated from changes in Hb and Hct (Costill et al., 1974). Fluid fluxes across the lung were calculated from plasma and EV changes. Fluid flux was quantified based on Q (Costill et al., 1974; Dill and Costill, 1974; Vengust et al., 2006a, 2013):

(1) $$J_{\text{PL}}=([{\text{PP}}_{\text{v}}]-[{\text{PP}}_{\text{a}}])/[{\text{PP}}_{\text{v}}])\text{ x }(1-\text{Hc}{\text{t}}_{\text{v}})\;\times \text{ Q}$$

for plasma fluid fluxes (*J*_PL_) where [PP_v_] is the plasma protein concentration in venous and [PP_a_] the plasma protein concentration in arterial blood, and

(2) $$J_{\text{ER}}=(([\text{H}{\text{b}}_{\text{v}}]/[\text{H}{\text{b}}_{\text{a}}])\text{ x }(\text{Hc}{\text{t}}_{\text{a}})-\text{Hc}{\text{t}}_{\text{v}})\;\times \text{ Q}$$

for erythrocyte fluid fluxes (*J*_ER_) where [Hb_v_] is Hb concentration in venous, [Hb_a_] Hb concentration in arterial blood, (Hct_v_) is Hct in venous and (Hct_a_) Hct concentration in arterial blood.

Fluid flux from or into the pulmonary vasculature was then calculated as the sum *J*_PL_ and *J*_ER_:

$$J_{\text{VA}}=J_{\text{PL}}+J_{\text{ER}}$$

Erythrocyte ion concentrations (_ER_[Ion]) were calculated from whole blood (_WB_) and plasma (_PL_) ion concentration according to Buono and Yeager (1986) and McKelvie et al. (1991).

(3) $${\;}_{\text{ER}}[\text{Ion}]={(}_{\text{WB}}[\text{Ion}]-{(}_{\text{PL}}[\text{Ion}]\;\;\times \;(1-\text{Hc}{\text{t}}_{\text{v}})))\;\;\times \text{ Hc}{\text{t}}_{\text{v}}^{-1}$$

Veno-arterial differences across the lung were corrected for ΔPV_VA_, ΔEV_VA_ and ΔBV_VA_ according to McKenna et al. (1997):

(4) $${\;}_{\text{PL}}[\text{Ion}{]}_{\text{VA}}=([\text{Ion}{]}_{\text{v}}/(1+(\Delta \text{PV}))-[\text{Ion}{]}_{\text{a}}$$

(5) $${\;}_{\text{ER}}[\text{Ion}{]}_{\text{VA}}=([\text{Ion}{]}_{\text{v}}/(1+(\Delta \text{EV}))-[\text{Ion}{]}_{\text{a}}$$

(6) $${\;}_{\text{WB}}[\text{Ion}{]}_{\text{VA}}=([\text{Ion}{]}_{\text{v}}/(1+(\Delta \text{BV}))-[\text{Ion}{]}_{\text{a}}$$

Erythrocyte electrolyte fluxes across the lung (*J*_ER_Ion) were calculated from changes in _ER_[Ion]_VA_, Hct_a_ and Q (Vengust et al., 2013):

(7) $$J_{ER}\text{Ion}{=}_{\text{ER}}\;[\text{Ion}{]}_{\text{V}-\text{A}}\times \text{Hc}{\text{t}}_{\text{a}}\;\times \text{ Q}$$

Whole blood electrolyte fluxes across the lung (*J*_WB_Ion) were calculated from changes in _WB_[Ion]_VA_ and Q:

(8) $$J_{\text{WB}}\text{Ion}{=}_{\text{WB}}\;[\text{Ion}{]}_{\text{VA}}\;\times \text{ Q}$$

### Statistical analysis

This was randomized double blind placebo controlled study. Mean and standard error (±SE) are reported for each variable. The data were analyzed with the random intercept mixed model. The preplanned differences were carried out with the contrast analysis, where *P*-values of the non-orthogonal contrasts were corrected with the Benjamini–Hocberg method for multiple comparisons. A *P*-value smaller than 0.05 was considered statistically significant. The computations were performed with R language for statistical computing (R version 3.0.3) (R Core Team, 2014).

## Results

All dogs were successfully recovered form anesthesia. No complications related to castration or ovariectomy were reported, nor were there any post-procedures adverse effect reported 6 months after the procedure.

### Cardiac output and pulmonary artery pressure

**Figure 1:**
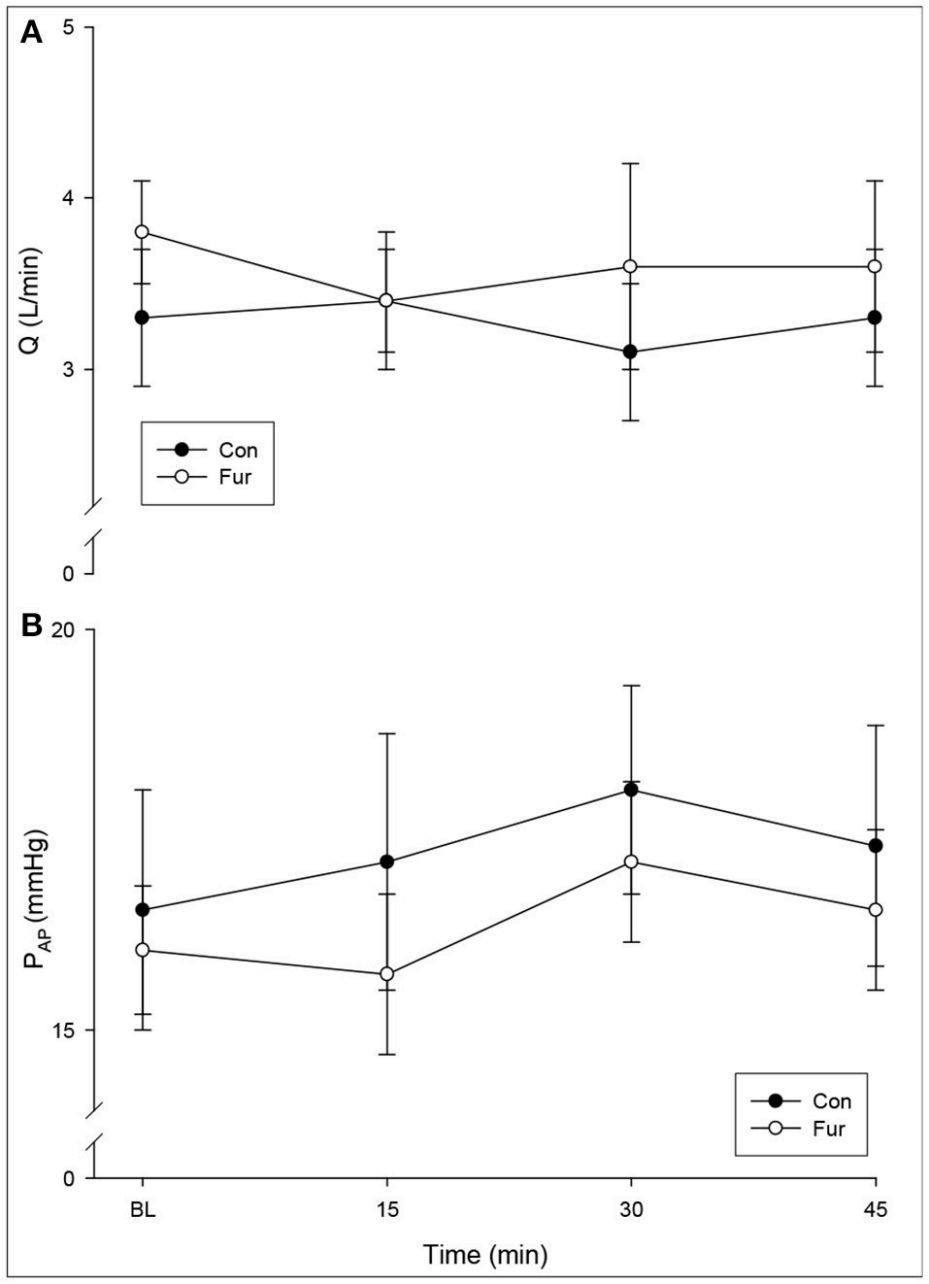
**(A)** Cardiac output (Q) and **(B)** mean pulmonary artery pressure (P_AP_) at Baseline (BL) and at 15, 30, and 45 min of general anesthesia. Values are means ± SE.

Cardiac output did not change with the duration of GA (time), nor was there a significant effect of Fur (BL: 3.3 ± 0.4 L/min in Con, 3.8 ± 0.3 L/min in Fur; 45 min: 3.3 ± 0.4 L/min in Con and 3.6 ± 0.5 L/min in Fur) (*P* = 0.5) (Figure 1A). Baseline P_AP_ was 15.5 ± 1.5 L/mmHg and 16.0 ± 0.8 mmHg in Con and Fur, respectively. There was only a slight increase in P_AP_ from baseline to 45 min in Con and Fur (*P* = 0.07) (45 min: 17.5 ± 1.4 mmHg in Con and 16.5 ± 1.0 mmHg in Fur) (Figure 1B).

### Haematocrit, hemoglobin, plasma protein, and blood gas difference across the lung

No effect of time on Hct_v_, Hct_a_, Hb_v_, Hb_a_, PP_v_, and PP_a_ was observed in Con, whereas Hct_v_, Hct_a_, PP_v_, and PP_a_, but not Hb_v_ and Hb_a_, increased significantly in Fur (*P* < 0.001) (Table 1).

**Table 1:** Respiratory changes across the lung, hemoglobin (Hb), Hematocrit (Hct), and Plasma protein (PP).

	**Treatment**	**BL**	**15 min**	**30 min**	**45 min**
**P**_v_**CO**_2_	Con	57.4 ± 3.2	59.2 ± 2.5	58.4 ± 1.3	57.4 ± 1.4
	Fur	59.5 ± 0.9	58.1 ± 2.1	56.5 ± 1.9	57.0 ± 1.1
**P**_a_**CO**_2_	Con	52.9 ± 2.1	54.0 ± 1.9	53.3 ± 1.5	52.6 ± 1.7
	Fur	54.6 ± 1.7	52.9 ± 2.0	50.0 ± 2.2	51.3 ± 1.8
Δ**C**_VA_**CO**_2_	Con	0.7 ± 0.7	0.3 ± 0.3	0.7 ± 0.4	0.5 ± 0.5
	Fur	1.2 ± 0.4	0.3 ± 0.4	1.2 ± 0.2	0.9 ± 0.3
**P**_v_**O**_2_	Con	83.6 ± 8.0	79.6 ± 5.1	78.1 ± 4.6	81.5 ± 5.0
	Fur	80 ± 4.4	72.6 ± 3.3	69.5 ± 3.0	68.1 ± 2.6
**P**_a_**O**_2_	Con	474 ± 13.2	484 ± 9.3	476 ± 12.8	477 ± 12.6
	Fur	479 ± 26.0	502 ± 15.1	511 ± 15.6	498 ± 12.9
Δ**C**_VA_**O**_2_	Con	3.6 ± 0.3	3.5 ± 0.3	3.8 ± 0.3	3.6 ± 0.5
	Fur^#^	3.4 ± 0.3	3.6 ± 0.4	4.1 ± 0.4^*^	4.4 ± 0.4^*^
**Hb**_v_	Con	13.9 ± 0.4	13.8 ± 0.3	13.5 ± 0.4	13.4 ± 0.5
	Fur^#^	13.2 ± 0.5	13.4 ± 0.5	13.9 ± 0.6^*^	14.0 ± 0.6^*^
**Hb**_a_	Con	14.2 ± 0.4	14.1 ± 0.4	14.1 ± 0.3	14.0 ± 0.3
	Fur^#^	13.5 ± 0.5	13.8 ± 0.6	14.3 ± 0.7^*^	14.4 ± 0.7^*^
^@^**PP**_v_	Con	59.0 ± 2.3	58.9 ± 2.2	58.5 ± 2.2	58.7 ± 2.1
	Fur^#^	60.8 ± 1.1	62.7 ± 1.2	65.2 ± 1.6^*^	66.4 ± 1.8^*^
^@^**PP**_a_	Con	58.8 ± 2.2	58.5 ± 1.9	59.3 ± 2.1	58.8 ± 2.0
	Fur^#^	60.6 ± 0.9	62.9 ± 1.1^*^	65.6 ± 1.5^*^	66.8 ± 1.5^*^
**Hct**_v_	Con	0.41 ± 0.01	0.41 ± 0.01	0.40 ± 0.01	0.41 ± 0.01
	Fur^#^	0.39 ± 0.01	0.39 ± 0.02	0.41 ± 0.02^*^	0.41 ± 0.02^*^
**Hct**_a_	Con	0.42 ± 0.01	0.42 ± 0.01	0.41 ± 0.01	0.41 ± 0.01
	Fur^#^	0.39 ± 0.01	0.41 ± 0.02	0.42 ± 0.02^*^	0.42 ± 0.02^*^

*All values are means ± SE. BL, Base line. PCO_2_, Blood CO_2_ partial pressure (mmHg) in arterial (P_a_CO_2_) and venous blood (P_v_CO_2_). PO_2_, Blood O_2_ partial pressure (mmHg) in arterial (P_a_O_2_) and venous blood (P_v_O_2_). ΔC_VA_CO_2_, Pulmonary CO_2_ veno-arterial difference (mmHg). ΔC_VA_O_2_, Pulmonary O_2_ veno-arterial difference (mmHg). Hb, hemoglobin in arterial (Hb_a_) and venous blood (Hb_v_) (g/L). Hct, hematocrit in arterial (Hct_a_) and venous blood (Hct_v_) (Proportion of 1.0). PP, plasma protein in arterial (PP_a_) and venous blood (PP_v_) (g/L). Con, Control. Fur, Furosemide treatment*.

# *Overall time effect significant*.

* *Different from BL*.

@ Fur effect significant

Arterial (P_a_O_2_) and venous (P_v_O_2_) blood O_2_ tensions were not affected by time or Fur. Similarly, arterial (P_a_CO_2_) and venous (P_v_CO_2_) blood CO_2_ tensions were not affected by time or Fur. Veno-arterial O_2_ difference (ΔC_VA_O_2_) increased with time in Fur (*P* = 0.001) from 3.4 ± 0.3 mmHg at BL to 4.1 ± 0.3 mmHg at 45 min. Veno-arterial CO_2_ difference (ΔC_VA_CO_2_) was not affected by time or Fur (*P* = 0.9) (Table 1).

### Volume and fluid changes across the lung

Baseline ΔEV was 0.6 ± 0.4% and 1.7 ± 0.7% in Con and Fur, respectively (*P* = 0.2), indicating a decrease in EV across the lung. Erythrocyte volume across the lung did not change with time (*P* = 0.9), and was not affected by Fur (*P* = 0.8). Baseline *J*_*ER*_ was 10.0 ± 6.0 and 25.0 ± 9.0 mL/min in Con and Fur, respectively (*P* = 0.1). In Con *J*_*ER*_ remained unchanged throughout the experiment, whereas in Fur at 15 min *J*_*ER*_ declined to 10.0 ± 3.0 mL/min (*P* = 0.03), and then returned to BL value (Table 2).

**Table 2:** Volume and fluid changes across the lung.

	**Treatment**	**BL**	**15 min**	**30 min**	**45 min**
Δ**EV**	Con	0.6 ± 0.4	1.3 ± 0.4	1.3 ± 0.2	1.3 ± 0.3
	Fur	1.7 ± 0.7	0.8 ± 0.3	0.8 ± 0.6	1.2 ± 0.4
Δ**PV**	Con	0.1 ± 0.5	−0.7 ± 1.0	1.5 ± 0.8	0.8 ± 0.9
	Fur	−0.3 ± 0.6	0.4 ± 0.5	0.7 ± 0.8	0.9 ± 1.0
Δ**BV**	Con	0.7 ± 0.4	0.8 ± 0.9	2.8 ± 0.9	2.1 ± 1.2
	Fur	1.4 ± 1.5	1.1 ± 0.6	1.5 ± 1.1	2.2 ± 0.5
***J**_*ER*_*	Con	10.0 ± 6.0	19.0 ± 6.0	17.0 ± 4.0	16.0 ± 5.0
	Fur	25.0 ± 9.0	10.0 ± 5.00^*^	16.0 ± 8.0	17.0 ± 9.0
***J**_*VA*_*	Con	3.0 ± 9.0	10.0 ± 11.0	37.0 ± 12.1	22.0 ± 17.0
	Fur	19.0 ± 21.0	17.0 ± 16.0	20.0 ± 22.0	34.0 ± 9.0

*All values are means ± SE. BL, Base line. ΔEV, Erythrocyte volume change across the lung (%).ΔPV, Plasma volume change across the lung (%). ΔBV, Blood volume change across the lung (%). J_ER_, Erythrocyte fluid flu across the lung (mL/min). J_VA_, Transvascular fluid fluxes across the lung (mL/min). Positive value indicates a net release of volume/fluid/ion from the compartment across the lung. Negative value indicates a net uptake of volume/fluid/ion from the compartment across the lung. Con, Control. Fur. Furosemide treatment*.

# *Overall time effect significant*.

* *Different from BL*.

@ *Fur effect significant*.

Baseline ΔBV were 0.7 ± 0.5% and 1.4 ± 1.5% in Con and Fur, respectively (*P* = 0.6), indicating a decrease in BV across the lung. Blood volume changes across the lung were not affected by time (*P* = 0.5) or Fur (*P* = 0.8). Baseline *J*_*VA*_ was 3.0 ± 9.0 and 19.0 ± 21.0 mL/min in Con and Fur, respectively (*P* = 0.5). Transvascular fluid fluxes remained unchanged over time (*P* = 0.4) and were not affected by Fur (*P* = 0.8) (Table 2).

### Ion changes across the lung

Plasma [H^+^] and [${\text{HCO}}_{3}^{-}$] changes across the lung were not affected by time or Fur (*P* ≤ 0.1) (Table 3).

**Table 3:** [H^+^] and [${\text{HCO}}_{3}^{-}$] changes across the lung.

	**Treatment**	**BL**	**15 min**	**30 min**	**45 min**
**[H**^+^**]**_a_	Con	54.5 ± 2.2	55.3 ± 2.0	55.2 ± 1.6	55.0 ± 1.3
	Fur	54.6 ± 1.8	54.0 ± 2.1	52.3 ± 2.2	53.4 ± 2.0
**[H**^+^**]**_VA_	Con	3.0 ± 2.0	5.4 ± 1.4	3.0 ± 1.2	3.4 ± 1.4
	Fur	4.1 ± 1.3	4.6 ± 0.8	3.8 ± 0.9	2.1 ± 0.9
**[${\text{HCO}}_{3}^{-}$]**_a_	Con	23.3 ± 0.3	23.3 ± 0.4	22.8 ± 0.4	22.8 ± 0.5
	Fur	24.0 ± 0.6	23.5 ± 0.5	23.0 ± 0.7	23.1 ± 0.7
**[${\text{HCO}}_{3}^{-}$]**_VA_	Con	−0.8 ± 14	−0.6 ± 1.2	1.0 ± 0.8	−0.01 ± 1.0
	Fur	−1.2 ± 1.2	0.2 ± 0.7	−0.2 ± 0.9	1.1 ± 1.1

All values are means ± SE. BL, Base line. [H^+^]_a_, Plasma hydrogen ion concentration in arterial blood. [H^+^]_VA_, Plasma hydrogen ion concentration difference across the lung (nmol/L). [${\text{HCO}}_{3}^{-}$]_V_, Plasma bicarbonate concentration in arterial blood. [${\text{HCO}}_{3}^{-}$]_VA_, Plasma bicarbonate concentration changes across the lung (mmol/L).Positive value indicates a net release of volume/fluid/ion from the compartment across the lung. Negative value indicates a net uptake of volume/fluid/ion from the compartment across the lung. Con, Control. Fur, Furosemide treatment. ^#^Overall time effect significant. ^*^Different from BL. ^@^Fur effect significant.

Baseline _*ER*_${\mathrm{Na}}_{VA}^{+}$ was −0.7 ± 0.3 mmol/L and −0.2 ± 0.3 mmol/L in Con and Fur, respectively (*P* = 0.4), indicating no or minimal increase in _*ER*_*Na*^+^. Throughout the experiment _*ER*_*Na*^+^ remained at BL levels in Con (45 min: −0.4 ± 0.7 mmol/L) and Fur (45 min:−0.6 ± 0.3 mmol/L), and was not affected by time (*P* = 0.8) or Fur (*P* = 0.9). Some increase in _*ER*_*Na*^+^ was evident in Fur at 30min (−1.2 ± 0.3 mmol/L); however, this value remained non-significant (*P* = 0.08). Baseline _PL_${\text{Na}}_{\text{VA}}^{+}$ was −0.7 ± 0.6 mmol/L and −0.9 ± 0.7 mmol/L in Con and Fur (*P* = 0.9), respectively, indicating a modest increase in _PL_Na^+^ across the lung. Throughout the experiment _PL_Na^+^ remained at BL level. Baseline _*WB*_${\mathrm{Na}}_{VA}^{+}$ was −1.5 ± 1.4 and −1.1 ± 0.9 mmol/L in Con and Fur, respectively (*P* = 0.9). Throughout the experiment _*WB*_*Na*^+^ continue to show a weak tendency to move into the vascular compartment in Con and Fur. Effects of time (*P* = 0.3) or Fur (*P* = 0.9) were not evident (Table 4).

**Table 4:** Sodium (Na^+^) changes across the lung.

	**Treatment**	**BL**	**15 min**	**30 min**	**45 min**
_ER_**${\text{Na}}_{\text{VA}}^{+}$**	Con	−0.7 ± 0.3	−0.5 ± 0.3	−0.6 ± 0.2	−0.4 ± 0.3
	Fur	−0.2 ± 0.3	−0.4 ± 0.6	−1.2 ± 0.3	−0.6 ± 0.7
_PL_**${\text{Na}}_{\text{VA}}^{+}$**	Con	−0.7 ± 0.6	−1.1 ± 1.2	0.8 ± 0.9	0.0 ± 0.0
	Fur	−0.9 ± 0.8	−0.3 ± 0.4	0.1 ± 0.8	1.2 ± 1.0
_WB_**${\text{Na}}_{\text{VA}}^{+}$**	Con	−1.5 ± 0.7	−1.6 ± 1.2	0.2 ± 0.9	−0.4 ± 1.1
	Fur	−1.1 ± 0.9	−1.1 ± 0.6	−1.1 ± 0.8	0.6 ± 0.6
***J***_ER_**Na**^+^	Con	−0.9 ± 0.5	−0.8 ± 0.4	−0.8 ± 0.4	−0.8 ± 0.4
	Fur	−0.2 ± 0.4	−0.8 ± 0.5	−1.6 ± 0.3	−0.7 ± 0.5
***J***_WB_**Na**^+^	Con	−2.8 ± 1.6	−2.9 ± 2.1	0.1 ± 1.3	−1.3 ± 2.9
	Fur	−2.4 ± 2.6	−1.3 ± 1.3	−2.4 ± 1.3	0.1 ± 2.3

All values are means ± SE. BL, Base line. _ER_${\text{Na}}_{\text{VA}}^{+}$, Erythrocyte Na^+^ changes across the lung. (mmol/L). _PL_${\text{Na}}_{\text{VA}}^{+}$, Plasma Na^+^ changes across the lung. _WB_${\text{Na}}_{\text{VA}}^{+}$, Whole blood Na^+^ changes across the lung. (mmol/L). J_ER_Na^+^ Erythrocyte Na^+^ flux across the lung (mmol/min). J_WB_Na^+^, Transvascular Na^+^ flux across the lung (mmol/min). Positive value indicates a net release of volume/fluid/ion from the compartment across the lung. Negative value indicates a net uptake of volume/fluid/ion from the compartment across the lung. Con, Control; Fur, Furosemide treatment. ^#^Overall time effect significant. ^*^Different from BL. ^@^Fur effect significant.

Baseline *J*_*ER*_*Na*^+^ was −0.9 ± 0.5 mmol/min and −0.2 ± 0.4 mmol/min in Con and Fur. Sodium erythrocyte fluxes remained unchanged over time in Con (45 min: 0.8 ± 0.4 mmol/min). In Fur with time _*ER*_*Na*^+^ influx increased to 1.6 ± 0.3 mmol/min at 30 min (*P* = 0.08), but then returned to BL vales at −0.7 ± 0.6 mmol/min at 45 min. Baseline *J*_*WB*_*Na*^+^ was −2.8 ± 1.6 mmol/min and −2.4 ± 1.8 mmol/min in Con and Fur (*P* = 0.9), respectively. Time (*P* = 0.4) and Fur (0.09) had no effect on *J*_*WB*_*Na*^+^ (Table 4).

Baseline _*ER*_$K_{VA}^{+}$ was −0.3 ± 0.2 and 0.4 ± 0.2 mmol/L in Con and Fur, respectively (*P* = 0.3). Time did not affect _*ER*_$K_{VA}^{+}$ in Con (*P* = 0.9), whereas in Fur _*ER*_*K*^+^ started to increase at 30 min (*P* = 0.03) and the returned to BL value at 45 min. Baseline _*PL*_$K_{VA}^{+}$ was −0.2 ±0.6 and −0.2 ±0.8 mmol/L in Con and Fur, respectively (*P* = 1.0). Although _*PL*_$K_{VA}^{+}$ started to decrease within plasma compartment with time in Con and Fur, this effect was not found to be significant (*P* = 0.3). Baseline _*WB*_$K_{VA}^{+}$ was −0.9 ±0.6 and −0.4 ±0.7 mmol/L in Con and Fur, respectively (*P* = 0.9). Time (*P* = 0.5) and Fur (*P* = 0.9) did not affect _*WB*_$K_{VA}^{+}$ (Table 5).

**Table 5:** Potassium (K^+^) changes across the lung.

	**Treatment**	**BL**	**15 min**	**30 min**	**45 min**
_ER_**${\text{K}}_{\text{VA}}^{+}$**	Con	−0.3 ± 0.3	−0.0 ± 0.2	−0.1 ± 0.2	−0.1 ± 0.2
	Fur	0.4 ± 0.2	−0.2 ± 0.2	−0.7 ± 0.2^*^	−0.1 ± 0.7
_PL_**${\text{K}}_{\text{VA}}^{+}$**	Con	−0.2 ± 0.6	−0.5 ± 1.0	1.5 ± 0.9	0.3 ± 1.2
	Fur	−0.2 ± 0.8	0.4 ± 0.5	0.7 ± 0.8	1.8 ± 0.7
_WB_**${\text{K}}_{\text{VA}}^{+}$**	Con	−0.9 ± 0.6	−1.0 ± 1.2	0.8 ± 0.9	0.4 ± 1.1
	Fur	−0.4 ± 0.7	−0.3 ± 0.6	−0.5 ± 0.8	0.2 ± 0.6
***J***_ER_**K**^+^	Con	−0.4 ± 0.3	−0.1 ± 0.4	−0.1 ± 0.4	−0.3 ± 0.2
	Fur	0.6 ± 0.3	−0.2 ± 0.3	−0.9 ± 0.3^*^	−0.1 ± 0.9
***J***_WB_**K**^+^	Con	−1.2 ± 1.2	−2.5 ± 1.4	2.0 ± 1.3	−0.4 ± 1.8
	Fur	0.1 ± 1.5	0.6 ± 1.3	−0.6 ± 1.4	2.2 ± 1.0

*All values are means ± SE. BL, Base line. _ER_${\text{K}}_{\text{VA}}^{+}$, Erythrocyte K^+^ changes across the lung. (mmol/L). _PL_${\text{K}}_{\text{VA}}^{+}$, Plasma K^+^ changes across the lung. _WB_${\text{K}}_{\text{VA}}^{+}$, Whole blood K^+^ changes across the lung. (mmol/L). J_ER_K^+^ Erythrocyte K^+^ flux across the lung (mmol/min). J_WB_K^+^, Transvascular K^+^ flux across the lung (mmol/min). Positive value indicates a net release of volume/fluid/ion from the compartment across the lung. Negative value indicates a net uptake of volume/fluid/ion from the compartment across the lung. Con, Control. Fur, Furosemide treatment. ^#^Overall time effect significant*.

* *Different from BL. ^@^Fur effect significant*.

Baseline *J*_*ER*_*K*^+^ was −0.4 ±0.3 mmol/min and 0.6 ±0.3 mmol/min in Con and Fur (*P* = 0.3), respectively. Potassium erythrocyte flux remained unchanged over time (*P* = 0.6) and was not affected by Fur (45 min: −0.3 ± 0.2 mmol/min in Con and −0.1 ± 0.9 mmol/min in Fur) (*P* = 0.9). Baseline *J*_*WB*_*K*^+^ was −1.2 ± 1.2 and 0.1 ±1.5 mmol/min in Con and Fur (*P* = 0.6), respectively. Potassium transvascular flux remained unchanged over time (*P* = 0.3) and was not affected by Fur (*P* = 0.3) (Table 5).

Baseline _*ER*_${\mathrm{Cl}}_{VA}^{-}$ was −0.4 ± 0.4 and 0.9 ± 0.3 mmol/L in Con and Fur, respectively (*P* = 0.7). Throughout the experiment _*ER*_*Cl*^−^ remained within BL values in Con (*P* = 0.8). In Fur at 30 min _*ER*_*Cl*^−^ efflux reversed to influx at −0.5 ± 0.4 mmol/L (*P* = 0.02). Overall effect of Fur was significant at *P* = 0.04. Baseline _*PL*_${\mathrm{Cl}}_{VA}^{-}$ was −0.3 ± 0.5 and 0.3 ± 0.6 mmol/L in Con and Fur, respectively (*P* = 0.9). In Con _*PL*_*Cl*^−^ efflux increased to 2.0 ± 0.8 mmol/L (*P* = 0.04) and returned o BL value by 45 min. Similar was not evident in Fur (*P* = 0.7). Baseline _*WB*_${\mathrm{Cl}}_{VA}^{-}$ was −0.7 ±0.8 and 1.3 ±0.7 mmol/L in Con and Fur, respectively (*P* = 0.2). With time _*WB*_*Cl*^−^ showed a weak tendency to efflux from the vascular compartment, which was most prominent at 30 min in Con (2.6 ± 0.7 mmol/L; *P* = 0.02). Similar was not evident in Fur (*P* = 0.7) (Table 6).

**Table 6:** Chloride (Cl^−^) changes across the lung.

	**Treatment**	**BL**	**15 min**	**30 min**	**45 min**
^@^**_ER_${\text{Cl}}_{\text{VA}}^{-}$**	Con	−0.4 ± 0.4	0.6 ± 0.3	0.5 ± 0.3	0.3 ± 0.3
	Fur	0.9 ± 0.2	0.3 ± 0.5	−0.5 ± 0.4^*^	0.2 ± 0.7
_PL_**${\text{Cl}}_{\text{VA}}^{-}$**	Con	−0.3 ± 0.5	0.1 ± 1.2	2.0 ± 0.8^*^	0.8 ± 1.0
	Fur	0.3 ± 0.6	0.8 ± 0.5	0.7 ± 0.9	1.2 ± 0.7
_WB_**${\text{Cl}}_{\text{VA}}^{-}$**	Con	−0.7 ± 0.8	0.7 ± 1.2	2.5 ± 0.7^*^	1.2 ± 1.1
	Fur	1.3 ± 0.7	1.1 ± 0.9	0.2 ± 1.1	1.5 ± 0.9
^@^***J***_ER_**Cl**^−^	Con	−0.2 ± 0.4	0.7 ± 0.4	0.8 ± 0.4	0.5 ± 0.3
	Fur	1.2 ± 0.4	0.6 ± 0.7	−0.6 ± 0.5^*^	0.4 ± 1.0
***J***_WB_**Cl**^−^	Con	−0.3 ± 1.2	−0.6 ± 1.8	3.5 ± 1.2	0.9 ± 2.2
	Fur	1.6 ± 2.5	1.8 ± 1.8	1.1 ± 1.7	2.9 ± 0.8

*All values are means ± SE. BL, Base line. _ER_${\text{Cl}}_{\text{VA}}^{-}$, Erythrocyte Cl^−^ changes across the lung. (mmol/L). _PL_${\text{Cl}}_{\text{VA}}^{-}$, Plasma Cl^−^ changes across the lung. _WB_${\text{Cl}}_{\text{VA}}^{-}$, Whole blood Cl^−^ changes across the lung. (mmol/L). J_ER_Cl^−^ Erythrocyte Cl^−^ flux across the lung (mmol/min). J_WB_Cl^−^, Transvascular Cl^−^ flux across the lung (mmol/min). Positive value indicates a net release of volume/fluid/ion from the compartment across the lung. Negative value indicates a net uptake of volume/fluid/ion from the compartment across the lung. Con, Control; Fur, Furosemide treatment. ^#^Overall time effect significant*.

* *Different from BL*.

@ *Fur effect significant*.

Baseline *J*_*ER*_*Cl*^−^ was −0.02 ± 0.4 mmol/min and 1.2 ± 0.4 mmol/min in Con and Fur (*P* = 0.1), respectively. Chloride erythrocyte flux did not change with time in Con, whereas in Fur at 30 min it changed to influx at 0.6 ± 0.4 mmol/min (*P* = 0.02). The overall effect of Fur on *J*_*ER*_*Cl*^−^ was significant at *P* = 0.05. Baseline *J*_*WB*_*Cl*^−^ was −0.3 ± 1.2 and 1.6 ± 2.5 mmol/min in Con and Fur (*P* = 0.4), respectively. In general, Cl^−^ showed the tendency to flux out of the vascular compartment throughout the experiment. In Con at 30 min *J*_*WB*_*Cl*^−^ was significantly different from BL (*P* = 0.04). No overall effect of Fur on *J*_*ER*_*Cl*^−^ was observed (*P* = 0.6) (Table 6).

## Discussion

This is the first report of fluid and ion fluxes across the pulmonary circulation during GA. In the present study we observed erythrocyte and blood volume changes across the lung (~1.0–1.5%), which are in line with those reported from horses at rest (Vengust et al., 2006a,b, 2011, 2013). Volume changes created *J*_*VA*_of ~20 mL/min. Dogs were treated with Fur to reduce hydrostatic forces and/or influence erythrocyte and transvascular ion metabolism (Mukherjee et al., 1981; Narins and Chusid, 1986; Boles Ponto and Schoenwald, 1990). Treatment with Fur caused dehydration and partially affected Cl^−^ metabolism across pulmonary vascular compartments; however, it did not change Q, P_AP_, and/or *J*_*VA*_. Our hypothesis was, therefore, verified.

### Effects of furosemide on *J_*VA*_*

Furosemide is used in patients with pulmonary edema. The reduction in lung water is due to a decrease in preload through venodilatation and diuresis (Dikshit et al., 1973), which decreases transmural hydrostatic pressures (Bake et al., 1968; Hlastala et al., 1996). Furosemide treatment in this study dehydrated dogs and caused volume depletion due to diuresis (Dikshit et al., 1973), which was not translated into reduced Q, P_PA_, and *J*_*VA*_. Transvascular fluid fluxes, however, were also not affected in previous studies where a significant decrease in Q due to Fur was reported (Wickerts et al., 1992; Vengust et al., 2011).

### Effects of general anesthesia on *J_*VA*_*

General anesthesia can cause some degree of ventilation perfusion (V/Q) mismatch (Gunnarsson et al., 1991), which is mitigated by a variable degree of HPV (Dueck et al., 1984). The most consistent triggering factor is the decrease in lung compliance and a fall in functional residual capacity (Mead and Collier, 1959; Bendixen et al., 1963). HPV optimizes systemic O_2_ delivery by constricting and increasing pressure in pulmonary microcirculation away from hypoxic lung regions (Madden et al., 1992). Increased pulmonary microvascular pressures change the balance between intra- and extravascular Starling forces and may influence *J*_*VA*_ (Starling, 1896; Vengust et al., 2013). In normal lungs, however, it is unlikely that the level of alveolar hypoxia during GA using normal concentration of volatile anesthetics would create edemagenic P_AP_ and clinical edema (Domino et al., 1986; Marshall et al., 1991). In the present study a steady but non-significant increase in *J*_*VA*_was observed within 45 min of GA on Con and Fur. This was equal in Con and Fur and, therefore, cannot be attributed to fluid therapy because of the diuretic effect of Fur (Mitchell et al., 1992). It is most likely that the supine (dorsal) position during GA was the reason for modest increase in *J*_*VA*_ (Wiener et al., 1990).

Opioid drugs, benzodiazepines and propofol used for premedication and induction of GA in this study do not affect pulmonary vascular reactivity, and are not considered a significant initiator for V/Q mismatch (Gibbs and Johnson, 1978; Benumof et al., 1987; Reves et al., 2010). Sevoflurane and other modern inhaled anesthetic on the other hand have a moderate inhibitory effect on HPV (Marshall et al., 1984; Wang et al., 1998; Kerbaul et al., 2006) and may even cause a reduction in *J*_*VA*_. Because sevoflurane in this study was delivered in O_2_, some degree of atelectasis would theoretically be expected (Sylvester et al., 2012). However, studies in animals and humans failed to generate significant O_2_ related shunt during GA (Wagner et al., 1974; Dantzker et al., 1975; Lundquist et al., 1988; Sylvester et al., 2012).

### Electrolyte and volume changes across the lung

In the present study Cl^−^ metabolism was the most affected by Fur, which coincided with the reduction of *J*_*ER*_ but did not influence *J*_*VA*_. Transvascular fluid fluxes in healthy individuals seem to be dependent on ΔEV (Vengust et al., 2006a, 2011, 2013). Erythrocytes have a complex and specific regulation of their volume through changes in their osmolality (van't Hoff, 1887; Hamburger, 1891, 1918). In peripheral tissues in deoxygenated blood, Cl^−^ (and water) is exchanged for ${\text{HCO}}_{3}^{-}$ across the erythrocyte plasma membrane (Hamburger, 1891, 1918; Bretcher, 1971). Na^+^/K^+^/2Cl^−^ cotransport across the erythrocyte plasma membrane is activated by similar stimuli and contributes to solute concentration in the erythrocyte. Erythrocyte osmolality persists at rather higher levels also due to lower PO_2_ in peripheral tissues, which inhibits K^+^/Cl^−^ cotransport/egress from erythrocytes. On contrary, the Na^+^/K^+^ ATPase activity across the erythrocyte plasma membrane decreases erythrocyte [Na^+^] and consequently erythrocyte osmolality. Na^+^/K^+^ ATPase effect, however, is inferior to combined activity of other ion channels, which work toward the increase of intracellular osmolality and EV. In the lung capillary bed increased PO_2_, efflux of Cl^−^, decreased [H^+^], and active K^+^/Cl^−^ cotransport across the erythrocyte plasma membrane reverse the process to erythrocyte regulatory volume decrease and fluid egress from erythrocytes (Fievet et al., 1990; Gibson et al., 1993, 1994, 2000; Honess et al., 1996; Speake et al., 1997; Juel et al., 1999). Previous studies in horses demonstrated, that *J*_*VA*_ is mostly dependent upon the Jacobs-Stewart cycle (Vengust et al., 2013), which is a cycle of intracellular-extracellular exchanges involving CO_2_, ${\text{HCO}}_{3}^{-}$, Cl^−^, and H^+^ across the erythrocyte membrane during capillary transit that speeds and enhances CO_2_ elimination (Jacobs and Stewart, 1942). As erythrocytes traverse the pulmonary microvasculature their membranes come into close contact with the capillary endothelium to form a functional single semi-permeable barrier. This semi-permeable “membrane” has the osmotic characteristics of the erythrocyte membrane itself (Hansen, 1961), and so may permit _*ion*_*J*_*VA*_ and *J*_*VA*_ (Vengust et al., 2013).

Increase in _ER_K^+^, and near significant increase of _ER_Na^+^ at 30 min indicated that Na^+^/K^+^/2Cl^−^ cotransport was affected by Fur. These changes were only detected across the erythrocyte membrane at 30 min of GA, which is consistent with furosemide pharmacokinetics in dogs (Hirai et al., 1992). However, changes in Cl^−^ metabolism across the lung were evident throughout the vascular compartment and not only across the erythrocyte membrane. These changes are not exclusive to Na^+^/K^+^/2Cl^−^ cotransport inhibition and should also be attributed to the Jacobs-Stewart cycle. Because Na^+^/K^+^/2Cl^−^ cotransport is also important at the vascular endothelial level where it contributes to the integrity of the permeability barrier (O'Donnell, 1993), the Jacobs-Stewart cycle assumed a transvascular role in maintaining the volume and ion equilibrium after Fur treatment.

### Other effects of furosemide relevant to pulmonary transvascular fluid fluxes

It would also be possible that Fur influences *J*_*VA*_ through other effects not directly related to diuresis. Furosemide causes direct pulmonary vasodilatation and improved pulmonary compliance, which should reduce the risk for *J*_*VA*_ (Lundergan et al., 1988; Silke, 1993; Greenberg et al., 1994) Hemodynamic properties of Fur are beneficial in patients with mild physical impairments due to ventricular dysfunction, whereas it seems that in healthy subjects are unlikely to show any quantifiable effect (Silke, 1993). Furosemide also induces a weak bronchodilator effect when inhaled in asthmatic humans (Bianco et al., 1988) or given intravenously to horses with (Rubie et al., 1993) or without the pulmonary obstructive disease (Olsen et al., 1992). Bronchodilation reduces the effect of exercise induced alveolar hypoxia and consequent pulmonary vasoconstriction of small pulmonary arteries, which increases pulmonary microvascular pressure and affects pulmonary capillary water permeability (Mairbäurl et al., 2002). The combination of Fur effect related to volume depletion, pulmonary vasodilatation and bronchodilation most probably contributed to better ΔC_VA_O_2_ in Fur in this study.

### Effects of [H^+^] on *J_*VA*_*

Changes in [H^+^] can influence vascular tone by regulating endothelium and vascular smooth muscle function (Aalkjaer, 1990). No acid base imbalance was noted in dogs in this study, which could potentially affect *J*_*VA*_. Alkalosis is consistently associated with the reduction in pulmonary microvascular pressures (Loeppky et al., 1985). Effects of acidosis, however, on pulmonary circulation vascular resistance is inconsistent. Pulmonary vasculature in general, unlike systemic circulation, shows resistance to vasodilator effect of extracellular acidosis (Aalkjaer, 1990; Barnes and Liu, 1995). Extracellular acidosis has also been shown to increase PVR in isolated dogs' pulmonary lobes, and calves and children with congenital heart disease and associated pulmonary hypertension (Lloyd, 1966; Rudolph and Yuan, 1966; Morray et al., 1988). Increase in pulmonary smooth muscle intracellular [H^+^], however, decreases PVR in isolated animal lungs (Raffestin and McMurtry, 1987; Ketabchi et al., 2009).

### Methodological considerations and limitations

The experimental methodology used in the present study has previously been validated (Costill et al., 1974; Dill and Costill, 1974; Vengust et al., 2006a, 2011, 2013). Variables measured are reproducible, have excellent CV and are able to detect small changes across different compartments. It is important to realize that methodology used herein enables an “*in-vivo*” investigation of lung fluid physiology. In contrast, lung lymph flow or pulmonary gravimetric techniques, two other methods to study lung fluid physiology, are more invasive, require post mortem examination, and/or require static investigation employing nuclear medicine. Lung lymph flow studies would also require better defined attention to the uncertainty concerning the tissues drained by the lymphatics and the effect of the lymph nodes themselves on lymph constituents (Coates et al., 1984; Newman et al., 1988). Gravimetric lung fluid dynamic studies only detect variations in the presence of lung water and are unable to account for alterations when changes are to be contributed to the vascular, interstitial, and/or cellular compartments in lungs (Lin et al., 1998; Hanel et al., 2003).

Most dog breeds have a very low Na^+^/K^+^ ATPase activity with consequent high erythrocyte Na^+^ and low erythrocyte K^+^ concentrations (Maede and Inaba, 1985). The importance of Na^+^/K^+^ ATPase activity with regards to *J*_*VA*_ is minimal, as discussed above. Reduced Na^+^/K^+^ ATPase causes erythrocyte Na^+^ and K^+^ concentrations to be similar to those in plasma, making intra-erythrocyte ion analyses in dogs less prone to an analytical error arising from high or low intracellular ion concentrations present in other species.

This study does not provide evidence and comparison between awake and anesthetized dogs. However, it is relevant to assume that *J*_*VA*_ in an awake dog is similar to BL values in this study (Wickerts et al., 1992; Vengust et al., 2006a). It is ethically unacceptable to instrument awake dogs in a manner such as used in this study, and physical restraint would cause a variety of stress related physiological changes.

## Conclusion

The dynamics of water movement in the pulmonary circulation are complex events encompassing Starling forces, gas exchange mechanisms, and EV regulation. Adaptations in ion metabolism in this study complimented the very low hydraulic conductance of the lung microvasculature, and prevented changes in *J*_*VA*_. The Jacobs Stewart cycle also seems to be an important safety factor for the stability of lung fluid dynamics. Differences in *J*_*VA*_ should be expected when alveolar epithelial and endothelial permeability are compromised due to e.g., mechanical ventilation and/or inflammation. Lung microvascular and alveolar permeability to proteins would then alter Starling forces and EV regulation and cause more prominent and abnormal lung fluid redistribution.

## Author contributions

MV, GH, and RB: participated in research design; OF, AD, AS, and MV: conducted experiments; OF, RB, GH, MV: performed data analysis. All authors wrote/contributed to the writing of the manuscript.

### Conflict of interest statement

The authors declare that the research was conducted in the absence of any commercial or financial relationships that could be construed as a potential conflict of interest.

## Abbreviations

Fur: Furosemide treatment
VA: Veno-arterial difference across the lung
*J*_VA_: Transvascular fluid fluxes in the pulmonary circulation
P_PA_: Pulmonary artery pressure
_*ion*_*J*_*VA*_: Transmembrane/transvascular ion fluxes
BL: Baseline
EV: Erythrocyte volume
PV: Plasma volume
*J*_PL_: Plasma fluid fluxes
*J*_ER_: Erythrocyte fluid fluxes
_ER_[Ion]: Erythrocyte ion concentrations
_WB_[Ion]: Whole blood concentrations
*J*_ER_Ion: Erythrocyte ion fluxes across the lung
*J*_WB_Ion: Whole blood fluxes across the lung
SID: Strong ion difference.
